# Brucellosis presenting as a spinal epidural abscess in a 41-year-old farmer: a case report

**DOI:** 10.4076/1757-1626-2-7614

**Published:** 2009-07-02

**Authors:** Ioannis Starakis, Katerina Solomou, Dimitrios Konstantinou, Chrysoyla Karatza

**Affiliations:** 1 Department of Internal Medicine, Patras University Hospital26500 Rion, PatrasGreece; 2 Department of Radiology, Patras University Hospital26500 Rion, PatrasGreece; 3 Department of Neurosurgery, Patras University Hospital26500 Rion, PatrasGreece

## Abstract

**Introduction:**

Brucellar epidural abscess is rare but potentially fatal medical entity and very few cases have been reported so far. Whilst in developed countries, cases of brucellar spondylitis and epidural abscess are unusual, since brucellosis has practically been eradicated in animals, in Greece it is one of the most frequent zoonosis. By reporting this case report we want to stress out the importance of early diagnosis and management and also that physicians should keep an open mind and high index of suspicion especially in regions where brucellosis is endemic or when their patients have traveled to these areas and have consumed unpasteurized dairy products.

**Case presentation:**

We present the case of a 41-year-old male Caucasian farmer complaining of acute, progressively worsening low back pain of five days duration. Fever with rigors, malaise and profuse night sweating were added to the symptoms, two days before admission. Magnetic Resonance imaging clearly showed the lesion and blood and tissue cultures were positive for *Brucella melitensis*.

**Conclusion:**

Spinal epidural abscess is a rare condition, difficult to diagnose, may be complicated by potentially disastrous neurological or vascular complications, and it can be fatal if left untreated. Patients complaining of fever and back pain, particularly in endemic areas should be investigated as possible cases of brucellosis and MRI is the method of first choice in the diagnostic process. Neurological dysfunction is often disproportionate to the observed degree of compression. A delay in diagnosis or surgical treatment may result in deleterious sequelae such as permanent paralysis or even death for patients with spinal epidural abscess.

## Case presentation

**Figure 1.**
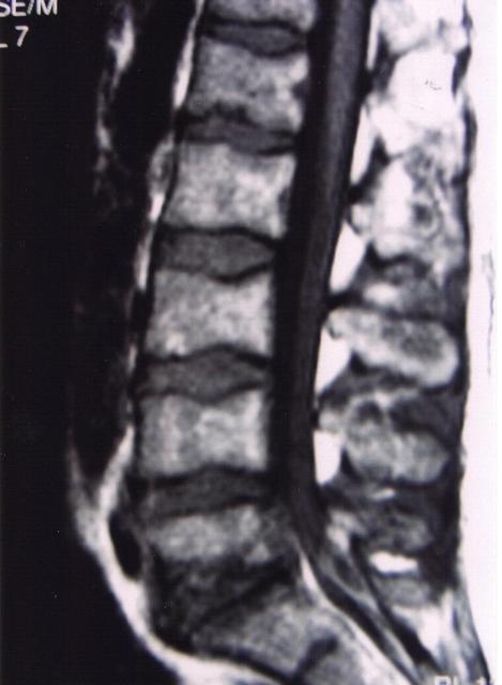
A sagittal pre-contrast T1-weighted spin-echo (SE) image shows a fluid collection in the posterior epidural space with low signal intensity, that is anteriorly compressing the dorsal sac and decreased signal intensity of the L5 and S1 vertebral bodies.

**Figure 2.**
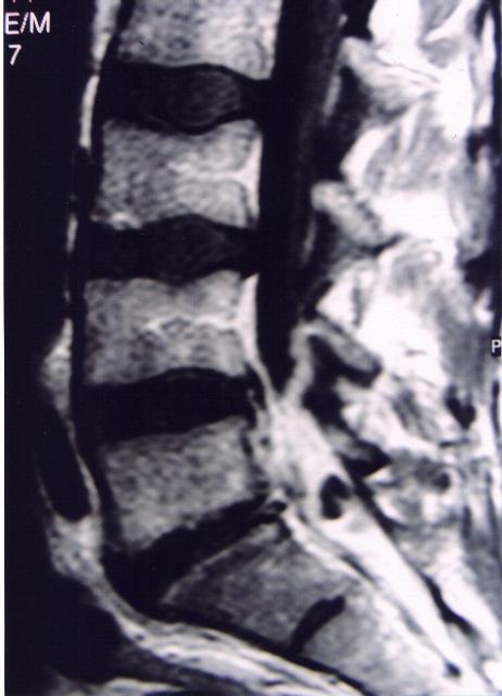
Following Gd administration, a sagittal T1-weighted image shows a marked enhancement of the L5 and S1 vertebral bodies, the L5-S1 inter-vertebral disk and the epidural fluid collection, suggesting spondylodiskitis with abscess formation at the L5-S1 level.

A 41-year-old male Caucasian farmer was admitted to our hospital complaining of acute low back pain of five days duration. Pain was progressively worsened and two days before admission fever with rigors, malaise and profuse night sweating were added to the symptoms. There was no past medical history. Plain films were unremarkable. A MRI study was performed. On the pre-contrast T1-Weighted Spin-echo (SE) sagittal images, a fluid collection in the posterior epidural space with low signal intensity and anteriorly compressing the dorsal sac was detected. A decreased signal intensity of the L5 and S1 vertebral bodies was also demonstrated (Figure 1). On the T2-Weighted SE sagittal sections, increased signal intensity of the fluid collection as well as the L5 and S1 vertebral bodies and the L5-S1 inter-vertebral disk were demonstrated. Following Gd administration, there was marked enhancement of the L5 and S1 vertebral bodies, the L5-S1 inter-vertebral disk and the epidural fluid collection, consistent with spondylodiskitis and abscess formation at the L5-S1 level (Figure 2). Pending the receipt of blood cultures’ results, meropenem and vancomycin intravenously were administered along with rifampicin and doxycycline per os. Fever subsided the next days but low back pain remained unchanged and it was intensified when patient’s right leg was elevated at 35°. On the 4^th^ hospital day, blood cultures revealed *Brucella melitensis*. Patient was operated on the 5^th^ hospital day and a broad L5 laminectomy was performed with complete abscess removal and decompression of the S1 nerve root. Patient’s post-operative course was uneventful and he was discharged seven days after surgery.

## Discussion

Spinal epidural abscess (SEA) is a rare condition, still remains a diagnostic challenge, may be complicated by potentially disastrous neurological or vascular complications, and it can be fatal if left untreated. Brucellosis, is a zoonosis with worldwide distribution, relatively frequent in South America and in Mediterranean countries. This systemic condition seldom produces spondylodiscitis, which in a minority of cases may be complicated by spinal epidural abscess. Spinal epidural abscesses account for 1 to 2 every 10000-hospital admissions, and *Staphylococcus aureus* is the agent most frequently implicated. Most cases of SEA occur in patients aged 30-60 years. Risk factors include immunocompromised states such as diabetes mellitus, alcoholism, chronic renal failure, cancer and acquired immunodeficiency syndrome, as well as spinal procedures including epidural anesthesia or analgesia and spinal surgery or trauma. No predisposing conditions are found in 20 percent of patients with SEA. Skin abscesses and furuncles represent the most common source of infection. SEA is primarily a bacterial infection, and the gram-positive *Staphylococcus aureus* is the most common causative agent, which accounts for about two-thirds of all cases [1]. *Brucella species* is responsible for only 0.1% of cases. The most common presenting symptoms of SEA are back pain and fever, and the lapse between the onset of pain and neurologic deficits is quite varied. Most epidural abscesses are located posteriorly in the thoracic and lumbar spine. Anterior SEA is usually associated with diskitis or vertebral osteomyelitis. Blunt trauma is reported to precede the appearance of SEA in 15 to 35 percent of cases. Neurologic dysfunction is often disproportionate to the observed degree of compression and a combination of compressive and ischemic effects may act in synergy to produce the deleterious sequelae of epidural abscess. Brucellosis, a zoonosis with a worldwide distribution, is a systemic infection caused by facultative intracellular bacteria of the genus *Brucella* that can involve many organs and tissues [2]. Magnetic resonance imaging (MRI) displays the greatest diagnostic accuracy and is the method of first choice in the diagnostic process [3]. Myelography, commonly used previously to diagnose SEA, is no longer recommended. Lumbar puncture to determine cerebrospinal fluid protein concentrations is not needed for diagnosis and entails the risk of spreading bacteria into the subarachnoid space with consequent meningitis; therefore, it should not be performed. Magnetic resonance imaging (MRI) is also the most sensitive radiologic technique to detect vertebral osteomyelitis. In one study of 103 patients, MRI showed either typical or suggestive changes of osteomyelitis in 91 percent of patients with symptoms of less than two weeks’ duration and in 96 percent of patients with symptoms of greater than two weeks’ duration [4]. Typical MRI findings in vertebral osteomyelitis include decreased signal intensity in the disk and adjacent vertebral bodies on T2-weighted images, loss of endplate definition on T1-weighted images, and contrast enhancement of the disk, adjacent vertebral bodies and involved paraspinal and paravertebral soft tissues on T1-weighted images [5]. Ring enhancement of paraspinal and epidural processes correlates with abscess formation, whereas homogeneous enhancement correlates with phlegmon formation. False-negative MRI findings have been reported rarely in patients with vertebral osteomyelitis and epidural abscess, especially in patients with concurrent meningitis or in patients with long linear abscesses without discrete margins [6]. Plain radiograms of the spine are rapid, inexpensive, and accessible, but their sensitivity is low in the first weeks of the disease. Because brucellar spondylitis is a slow process, changes in plain radiographs can be difficult to differentiate from those of degenerative diseases [7]. Radionuclide scintigraphy although it is well suited for total-body assessment of the extent and distribution of musculoskeletal involvement [7], the limited tissue resolution of scintigraphy may necessitate the use of additional imaging modalities such as MRI. The therapeutic method of choice is laminectomy and drainage of the abscess combined with antibiotics. Conservative treatment alone is justifiable only for specific indications. Laminotomy is a therapeutic alternative for children. Parallel to improvements in the mortality rate, today more patients experience complete recovery from SEA. Our patient had no predisposing conditions for SEA but he daily consumed unpasteurized milk.

## Conclusions

Brucellar epidural abscess occurs rarely but represents a neurosurgical emergency because of its potential for causing rapidly progressive spinal cord compression and permanent paralysis. Family physicians, emergency department personnel as well as infectious diseases specialists should always keep a high index of suspicion and include brucellosis in their diagnostic evaluations. Furthermore, incisive monitoring by the attending physicians and critical care nurse may improve patient outcome through early recognition and intervention.

## Abbreviations

MRI: magnetic resonance imaging
SE: spin-echo
SEA: spinal epidural abscess
